# Does Venture Capital Backing Improve Disclosure Controls and Procedures? Evidence from Management’s Post-IPO Disclosures

**DOI:** 10.1007/s10551-022-05272-1

**Published:** 2022-11-06

**Authors:** Douglas Cumming, Lars Helge Hass, Linda A. Myers, Monika Tarsalewska

**Affiliations:** 1 Florida Atlantic University, Boca Raton, FL USA; 2 University of Strathclyde, Glasgow, UK; 3 University of Tennessee, Knoxville, Knoxville, TN USA; 4 University of Exeter Business School, Exeter, UK; 5 Birmingham Business School, University of Birmingham, Birmingham, UK

**Keywords:** Management disclosure, Material weaknesses, Disclosure controls and procedures, Venture capital, Initial public offerings, Sarbanes–Oxley Act of 2002, Corporate governance

## Abstract

Firm managers make ethical decisions regarding the form and quality of disclosure. Disclosure can have long-term implications for performance, earnings manipulation, and even fraud. We investigate the impact of venture capital (VC) backing on the quality and informativeness of disclosure controls and procedures for newly public companies. We find that these controls and procedures are stronger, as evidenced by fewer material weaknesses in internal control under Section 302 of the Sarbanes–Oxley Act, when companies are VC-backed. Moreover, these disclosures are informative and are more likely to be followed by subsequent financial statement restatements than are disclosures made by non-VC-backed IPO companies.

## Introduction

Disclosure is widely regarded as a central tenet of business ethics research. Prior research finds that disclosure can mitigate information asymmetry and improve market efficiency and fairness, leading to more equitable outcomes.^1^ High-quality disclosure at the time of an initial public offering (IPO) is critical because investors face the classic “lemons” problem (Akerlof, 1970) due to information asymmetry and agency frictions (Colombo et al., 2018; Scarlata & Alemany, 2010). Prior research finds that underwriters and venture capitalists (VCs) act as gatekeepers and monitors, helping to mitigate the lemons problem (Wongsunwai, 2013). Yet, scandals reported in the media reveal that even highly reputable investment banks and VCs support low-quality investments, and can use selective disclosure to mislead potential investors.^2^ In this paper, we address an important question because we study the role of VC investors in their investee firms’ disclosure decisions.

Venture capital (VC) funds provide financing to entrepreneurial companies before they go public. They can also reduce information asymmetries between managers and other investors by certifying the quality of companies in their portfolios via their advisory and monitoring roles (Gompers & Lerner, 1999; Manigart & Wright, 2013; Ritter, 2015). In order to perform these roles, VCs use disclosures provided by company management to make periodic reports to investors who committed capital to the VC fund (Johan & Zhang, 2021).

We ask whether VC backing improves financial disclosures made by newly public companies by analyzing the relation between VC backing and the quality of disclosure controls and procedures under Section 302 of the Sarbanes–Oxley Act of 2002 (SOX). In this way, we provide new evidence on whether VC firms can help to mitigate information asymmetry problems between managers and investors around the time of an IPO by improving the disclosure of material weaknesses in investee company controls.

Disclosure quality is an important ethical issue because high-quality disclosure fosters transparency in financial markets. In fact, one of management’s key responsibilities is providing important disclosure to stakeholders. To do this, managers must design and establish processes and safeguards over financial reporting that mitigate financial reporting risks (SEC 2002; CAQ 2013). Under Section 302 of SOX, the chief executive officer (CEO) and chief financial officer are directly responsible for the quality of the financial reports, as well as for establishing, maintaining, and evaluating the effectiveness of internal controls over financial reporting.^3^ Management is also required to report on their conclusions about the effectiveness of internal controls and to disclose any material weaknesses.^4^

High-quality internal controls should also be important to VC firms because disclosure is a key element of efficient VC investment. Internal controls improve the quality of the financial information used by VC firms to advise and monitor management, and because high-quality disclosure controls and procedures enhance investor confidence in companies’ financial reports, allowing VC firms to earn higher capital gains upon exit (Fassin & Drover, 2017; Hain et al., 2016; Scarlata et al., 2017). In addition, by improving the quality of investee company internal controls, VC firms can enhance their reputation as value-added active investors, allowing them to attract future deals from other entrepreneurs, syndicated investors, and institutional investors (Nahata, 2008).

Disclosure policies are effective when the information produced is incorporated in the decision-making processes of information users (Weil et al., 2006). According to a white paper authored by the Working Group on Director Accountability and Board Effectiveness of the National Venture Capital Association, “[w]hile private companies are not subject to most aspects of Sarbanes Oxley and other regulations that impose governance standards upon public companies, it is in the best interests of the venture capital industry to develop and proactively follow financial reporting best practices in the private company context” (NVCA 2007, p. 3). We therefore expect VC expertise and knowledge to support the establishment of high-quality disclosure controls and procedures, and we expect VC firms to demand that management invest in strong internal control systems because these systems facilitate VC decision-making and improve the VC firms’ ability to monitor their investments.^5^ That is, if VC firms incorporate information from management into their decisions, they have incentives to require that their investee companies establish strong internal control systems because internal control quality affects the information environment.^6^

Using data on the quality of internal controls from 2002 through 2018 for a sample of 694 US IPOs and econometric techniques that account for endogenous selection related to VC backing, we investigate whether VC-backed companies are more or less likely than non-VC-backed companies to disclose material weaknesses in disclosure controls and procedures under Section 302 of SOX post-IPO. In further analyses, we test whether the shareholdings of VC companies impact the relation between VC backing and the quality of internal controls. We also test whether VC backing differentially affects account-level material weaknesses, which relate to the application of specific accounting rules, versus entity-level material weaknesses, which are more pervasive and could relate to issues like unethical “tone at the top.” Finally, we test whether these material weakness disclosures are informative for market participants and whether VC-backed companies make disclosures that are more informative about future financial statement restatements. This latter test is important because these restatements provide evidence that the financial disclosures managers previously made to their stakeholders were materially misstated.

We find that companies with VC backing are less likely to report material weaknesses in disclosure controls and procedures in the three years post-IPO. We suggest that VC investee companies’ focus on providing a strong internal control environment could explain why Wongsunwai (2013) finds less earnings management and fewer financial statement restatements among VC-backed companies. When we distinguish between account-level and entity-level material weaknesses, we find that VC-backed companies are less likely to report both types. Therefore, VC backing is associated with better disclosure controls and procedures at the overall company level (including the “tone at the top,” separation of duties, etc.) and when related to specific accounting transactions. Finally, we find that a material weakness in year *t* is more likely to be followed by a restatement of the year *t* financial statements when the company is VC-backed. This is important because it provides evidence that disclosures about material weaknesses in internal control are more credible for companies backed by VC investors. Therefore, VC backing can support the main goal of disclosures—building trust in financial markets. Overall, we find pervasive evidence that investments by VC firms are associated with improved disclosure controls and procedures and with more informative internal control disclosures.

This paper contributes to several streams of literature. First, it contributes to research on the importance of disclosure and the conditions under which high-quality, effective disclosure is informative to financial markets and is, therefore, a central part of research in business ethics (Aitken et al., 2015; Baudot et al., 2021). Prior research suggests that not all disclosures are ethical and managers can make strategic disclosures that mislead investors (Blanc et al., 2019; Du, 2014; Fasterling, 2012; Laufer, 2003; Weil et al., 2006). We contribute to this literature by showing that internal control disclosure is more informative for financial markets in the case of VC-backed firms.

Second, it contributes to research on the role of VC backing in establishing and maintaining sound financial reporting practices and improving trust in financial markets. Building on prior research, which finds that VC firms constrain earnings management in newly public companies (Lee & Masulis, 2011; Morsfield & Tan, 2006; Nam et al., 2014; Wongsunwai, 2013), our focus is on whether VC backing impacts the quality of investee firms’ disclosure controls and procedures. Our finding that VC-backed companies have stronger internal control systems suggests a mechanism by which VC firms might constrain earnings management and reduce information asymmetry problems between investors and newly public companies.

Third, we extend prior research which suggests that due diligence, financial reporting quality, accounting disclosures, and building trust all play important roles for companies backed by financial intermediaries. For example, Cumming and Zambelli (2017) finds that better due diligence results in higher investee performance, and de Carvalho et al. (2020) shows that financial statement information plays an important role in the valuation of VC-backed companies before they are taken public. Beuselinck et al. (2008) finds that the quality of accounting information decreases with private equity ownership, and Beuselinck and Manigart (2007) documents an increase in disclosure following private equity investments. In contrast, we find that the quality of disclosure controls and procedures varies with VC backing. This is important because high-quality internal controls increase the reliability of the information disclosed in the financial statements and improve trust in financial markets.

The remainder of this paper is organized as follows. The next section discusses prior literature and develops our empirical prediction. Thereafter, we describe our empirical methodology, including our sample selection process and research design. Empirical tests are presented next along with a discussion of the results. The final section concludes.

## Prior Literature and Empirical Prediction

### The Role of Venture Capital Pre-IPO

VC firms are specialized financial intermediaries that are typically organized as limited partnerships with investment horizons of approximately 10 years. They source funds from a variety of institutional investors including pension funds, endowments, and life insurance companies, and they invest those funds in private early-stage entrepreneurial companies, which typically have scant assets and collateral (Mayer et al., 2005).

VC funds are generally not well-diversified and VC firms typically add only three or fewer investments per managing partner per year, until the fund’s committed capital is fully invested (Kanniainen and Keuschnigg 2013). Because VC firms profit from capital gains on their equity investments, they have strong incentives to actively monitor their investee companies over their entire investment horizons. These investments typically begin three to five years prior to the investee’s IPO or acquisition (Ritter, 2015) and their horizons typically extend well beyond the standard initial lockup period of 180 days post-IPO (Field & Hanka, 2001; Gompers & Lerner, 1999).

VC firms can also assist with contract design to mitigate agency problems (Scarlata & Alemany, 2010). For example, VC firms put in place compensation contracts that align CEO incentives with their own, and this increases valuation at the time of the IPO (Cadman and Sunder 2014). VC firms also negotiate with entrepreneurs to create systems of cash flow rights and monitoring rights that are contingent on company performance (Caselli et al. 2013). The contracts established by VC funds affect the VC-entrepreneur relationship by improving transparency and reducing agency problems (Gompers & Lerner, 1999).

VC monitoring and advising has profound effects on portfolio companies. For example, VC-backed companies have higher rates of innovation (Bertoni et al., 2011), receive higher purchase prices in acquisitions (Masulis & Nahata, 2011), enjoy improved performance and higher survival rates (Krishnan et al., 2011; Nahata et al., 2014), and experience lower rates of fraud (Tian et al., 2016). These performance benefits are compounded when VC firms have high reputations (Huang et al., 2016).

Previous evidence based on small samples of VC firms suggests that they can affect financial disclosure through direct control. For example, if the investee company does not perform well or if VC firms observe irregularities, they can take full control of the company and replace its management with managers selected by the VC firm.^7^ Therefore, it is reasonable to expect that if poor financial disclosures negatively affect firm value, VC firms can take control of the company and replace management.

The presence of VC investors should improve disclosure controls and procedures related to financial reporting because VC firms rely on information from their investee companies’ reporting systems to secure successful exit. Consistent with this, Davila and Foster (2005, 2007) finds that companies with VC investors are more likely to adopt formal management accounting systems, and Bernstein et al. (2016) provides evidence that VC firms’ on-site involvement in their portfolio companies increases the likelihood of a successful exit.

### Ethics and Disclosure

Disclosure is an important part of socially responsible behavior and corporate responsibility (Botosan, 1997; Lang & Lundholm, 1996; Gelb & Strawer, 2001). Schipper (1989) explains that partial communication, especially in the presence of asymmetric information, enables managers to engage in unethical behaviors such as earnings manipulation. Gelb and Strawer (2001) argues that by providing informative disclosure, firms are engaging with stakeholders. Ethical reporting is perceived as socially responsible activity and benefits stakeholders through increased transparency and enables better monitoring. Jensen and Meckling (1976) highlights that strong monitoring by outside shareholders is needed to reduce agency costs arising from the separation of ownership and control.

Prior research suggests that ethics and disclosure are interlinked, and disclosure coupled with ethical behavior can have a lasting positive effect on performance (Jo & Kim, 2008). Ineffective disclosure control systems increase the likelihood of corporate fraud, which is costly for shareholders and management alike (Karpoff et al., 2008a, 2008b). Prior research also finds that ineffective disclosure controls increase the likelihood of financial statement misstatements (Doyle et al., 2007), lead to auditor resignations (Hammersley et al. 2012), and are associated with lower management compensation (Hoitash et al., 2012) and less profitable investment decisions (Harp & Barnes, 2018). Therefore, manager and owner decisions about disclosure have long lasting consequences for future decisions related to earnings manipulation and performance.

### Venture Capital Backing and Disclosure

The effectiveness of the internal control system and disclosures about the system’s effectiveness are ethical decisions that have long-term consequences. High-quality control systems are crucial for providing high-quality financial information, and this information is critical for monitoring and forecasting future company performance (Feng et al., 2009) and when estimating the future profitability of investment opportunities (Feng et al., 2015; Harp & Barnes, 2018), as well as for successful VC firm exits. Prior research shows that investors value information about the strength of internal controls because market reactions are negative when companies exclude certain operations from their internal control audits (Carnes et al. 2019). Moreover, Hammersley et al. (2008) documents negative stock price reactions to the disclosure of material weaknesses in disclosure controls and procedures under Section 302 of SOX.

Institutional investors can affect their investee companies’ disclosure in numerous ways. Li et al. (2021) finds that mutual funds focused on corporate social responsibility can affect these disclosures. Similarly, Flammer et al. (2021) shows that investor activism influences firms’ voluntary disclosure of climate risks. In contrast to other institutional owners, VC firms make a small number of non-diversified investments and therefore have stronger incentives to make improvements at their investee companies. Venture capitalists sit on their investee company boards of directors (Amornsiripanitch et al., 2019; Gompers & Lerner, 1999) and regularly meet with senior management to discuss accounting, financing, recruitment, strategy, and valuation (Gorman & Sahlman, 1989). In addition, VC firms also have strong incentives to be effective monitors (Tian et al., 2016). Thus, although VC firms invest in private companies that are not subject to the requirements of SOX, we argue that they have incentives to establish strong internal controls and to improve disclosure systems in their investee companies. This allows VCs to monitor entrepreneurs, who may pursue their non-pecuniary personal interests at the expense of maximizing the overall firm value (Aghion & Bolton, 1992; Broughman, 2010), and to maximize value upon exit (Cumming, 2008).

First, in order to act as effective monitors, VC firms must have access to reliable financial information about the companies in their portfolios. Ineffective disclosure controls and procedures lead to low-quality financial reporting and introduce information uncertainty, which can adversely affect VC firms’ ability to make sound decisions. Second, VC firms often provide financing to companies whose ability to access subsequent financing is conditional on performance. Also, VC firms aim to maximize investee profitability and thus stock price at the time of exit in order to achieve the best exit strategy. Weaknesses in internal controls and disclosure hinder VC firms’ ability to access reliable information about company performance, which negatively impacts their ability to monitor and support their investee companies. Holder-Webb and Cohen (2007), Jo and Kim (2008), and Baudot et al. (2021), among others, consider the effect of disclosure on firm risk and performance, as well as the importance of disclosure for the profitability of VC investments. In summary, because VC firms have strong incentives to require that investee companies provide high-quality financial disclosure in order to maximize stock price at the time of exit, we expect VC-backed companies to have better internal control systems and disclosures.

Disclosure is important for fostering transparency in financial markets but compliance with disclosure regulations relies on managers making ethical choices. Managers can comply with established regulations or may fail to comply when they expect that noncompliance will not to be detected. The triangle theory suggests that noncompliance can occur when individuals have (1) opportunity, (2) attitude, and (3) incentives (Cressey, 1950; Trompeter et al., 2012). In theory, VCs might therefore allow for the non-disclosure of material weaknesses because of short-term benefits at the time of IPO. However, in the long term, repeated interactions between VCs, investors, financial institutions, etc., make this unlikely because VC reputation is valuable, and if VCs allow for “anti-disclosure bias” in the short term, they should be penalized in the long term. Consistent with this, VC firms that fail to prevent fraud in their portfolio companies are penalized by the market (Tian et al., 2016), and because material weaknesses are typically followed by restatements, the costs associated with undisclosed material weaknesses could outweigh any benefits of non-disclosure. Overall, we predict that VC-backed companies will make more informative material weakness disclosures than non-VC-backed companies, and that VCs should not allow for an anti-disclosure bias in the portfolio companies.

## Empirical Methodology

### Sample Selection

We collect information on whether the company was backed by a VC firm from the SDC Thomson One database and from Venture Xpert. Specifically, to select our IPO sample, we follow previous studies and identify transactions in SDC Thomson One where the “original IPO” flag is set to “yes.” We require that the marketplace be “US Public” and that the type of security be “Common Shares.” We eliminate financial institutions, real estate investment trusts, closed-end funds, spin-offs, former leveraged buyouts, and foreign companies because the disclosures made by these companies are quite different from disclosures made by other companies. We collect IPO-related information from SDC Thompson One. Next, we follow Chemmanur et al. (2018) and confirm that these IPOs are VC-backed using Venure Xpert. We also use Venure Xpert to access information about VC characteristics. Our sample consists of all IPOs from 2002 through 2018 that meet our selection criteria. Our tests require data on internal control quality for three years post-IPO, so we eliminate companies that were acquired within three years of going public. We also require financial data from Compustat. The sample composition is described below.

We obtain information on the strength of disclosure controls and procedures, as reported by management in filings with the US Securities and Exchange Commission under SOX Section 302, from the Audit Analytics database. Section 302 provides management’s assessment of the strength of internal controls on a quarterly basis and IPO companies must provide this evaluation from the first quarter following the IPO onward.^8^ Studies focusing on internal control quality at mature companies also consider annual disclosures made by management and the external auditors under SOX Section 404 but newly public companies are exempt from these reporting requirements until their second 10-K filing post-IPO. Moreover, the Jumpstart Our Business Startups Act of 2012 exempts newly public companies from complying with SOX Section 404 for up to five years if they qualify as emerging growth companies.

Following many prior studies, we focus on material weaknesses in internal control (rather than significant deficiencies or control deficiencies) because material weaknesses are the most severe form of internal control weakness (SEC, 2003) and most negative consequences documented in prior literature relate to material weaknesses. Implicit in our tests, and in most prior research, is the assumption that companies not reporting material weaknesses have effective disclosure controls and procedures (i.e., any weaknesses in their internal controls are at worst significant deficiencies, which do not have to be disclosed under SOX Section 302). As we explain above, VC firms have strong reputational and economic incentives to ensure that this is the case for their investee companies.

Table 1, Panel A summarizes our sample construction. Our final sample consists of 694 IPO companies: 483 (70%) are VC-backed and 211 (30%) are non-VC-backed. These proportions are consistent with the sample in Wongsunwai (2013) and our sample statistics are consistent with those reported in Ritter (2014a). Panels B through D reveal that the sample represents a variety of industries, US regions, and years, respectively.

**Table 1:** Sample selection and sample composition

Panel A: Sample selection		
Screening criteria	*N*	*N*
SDC Thomson One Sample from 2002 through 2018		19,670
*Less*		
Marketplace is not equal to US Public	4,293	
Type of security is not equal to Common Shares	11,251	
Real estate investment trusts	175	
Former leveraged buyouts	7	
Closed-end funds	705	
Spin-offs	448	
Data missing after merging with Venture Xpert	10	
Missing Compustat data	861	
Missing Audit Analytics data (2002 through 2019)	629	
Missing CRSP data	32	
Missing independent variables required for the tests	542	
Financial companies	23	
Final sample		694

Panel B: Sample composition by industry		
Industry Description (Fama–French 12 classifications)	*N*	%
Consumer Non-Durables	21	3.03
Consumer Durables	14	2.02
Manufacturing	43	6.20
Oil, Gas, and Coal Extraction	36	5.19
Chemicals and Allied Products	15	2.16
Business Equipment	229	33.00
Telephone and Television Transmission	21	3.03
Utilities	6	0.86
Wholesale, Retail, and Some Services	57	8.21
Healthcare, Medical Equipment, and Drug	192	27.67
Other	60	8.65
Total	694	100.00

Panel C: Sample composition by state		
State	*N*	%
Alabama	1	0.14
Arizona	14	2.01
California	204	29.35
Colorado	20	2.88
Connecticut	6	0.86
District of Columbia	3	0.43
Delaware	1	0.14
Florida	16	2.30
Georgia	20	2.88
Iowa	4	0.58
Idaho	3	0.43
Illinois	27	3.88
Indiana	9	1.29
Kansas	2	0.29
Kentucky	5	0.72
Louisiana	4	0.58
Maryland	72	10.36
Massachusetts	16	2.30
Michigan	6	0.86
Minnesota	13	1.87
Missouri	3	0.43
Montana	1	0.14
Nebraska	15	2.16
Nevada	1	0.14
New Hampshire	1	0.14
New Jersey	19	2.73
Nevada	8	1.15
New York	31	4.46
Ohio	6	0.86
Oklahoma	7	1.01
Oregon	4	0.58
Pennsylvania	26	3.74
South Carolina	1	0.14
South Dakota	1	0.14
Tennessee	6	0.86
Texas	72	10.36
Utah	7	1.01
Virginia	19	2.73
Washington	12	1.73
Wisconsin	6	0.86
Wyoming	2	0.29
Total	694	100.00

Panel D: Sample composition by year						
Year	VC-backed		Non-VC-backed		All IPOs	
	*N*	%	*N*	%	*N*	%
2002	4	0.83	1	0.47	5	0.72
2003	17	3.52	10	4.74	27	3.89
2004	46	9.52	25	11.85	71	10.23
2005	35	7.25	27	12.80	62	8.93
2006	44	9.11	29	13.74	73	10.52
2007	57	11.8	21	9.95	78	11.24
2008	3	0.62	7	3.32	10	1.44
2009	15	3.11	11	5.21	26	3.75
2010	28	5.80	9	4.27	37	5.33
2011	26	5.38	10	4.74	36	5.19
2012	26	5.38	12	5.69	38	5.48
2013	45	9.32	11	5.21	56	8.07
2014	49	10.14	7	3.32	56	8.07
2015	30	6.21	4	1.90	34	4.90
2016	21	4.35	9	4.27	30	4.32
2017	16	3.31	9	4.27	25	3.60
2018	21	4.35	9	4.27	30	4.32
Total	483	100	211	100	694	100.00

### Setting and Variable Descriptions

#### Disclosure Controls and Procedures

Management’s fiduciary duties under SOX Sections 302 and 404 involve establishing and maintaining good quality internal control systems as well as identifying and disclosing deficiencies or weaknesses that could result in material financial statement errors. Management must provide Section 302 and 906 certifications on Form 10-K and Form 10-Q starting with their first filing after going public.^9^ SOX Section 302 requires top management to: (i) establish and maintain a system of internal controls, (ii) design these internal controls to ensure that management receives any material information, (iii) evaluate the effectiveness of the internal controls, (iv) identify and disclose all significant deficiencies and material weaknesses in the design or operation of the internal controls to the audit committee and the external auditors, and (v) disclose any fraud involving employees with a significant role in the internal control system.^10^ Furthermore, SOX Section 404 establishes rules regarding the assessment of internal controls and specifies the role that management plays in establishing an adequate internal control system and effective internal control procedures. Finally, Section 906 aligns the incentives of top executives with those of shareholders by imposing criminal penalties (of up to $5,000,000 and/or imprisonment for up to 20 years) if the reported information is not compliant. Details of any material weaknesses are usually provided in *10-K report Item 9A: Controls and Procedures*.

A variety of problems can generate material weaknesses in disclosure controls and procedures. Control problems can include account-level (accounting-related) problems as well as entity-level problems. Account-level material weaknesses indicate that management’s assessment of internal controls has identified an accounting rule application failure. For example, the Audit Analytics category *Capitalization of Expenditures Issues* consists of internal control deficiencies in approach, theory, or calculation associated with the capitalization of expenditures. These can include expenditures capitalized for inventory, construction, intangible assets, research and development, software or product development, and other purposes.^11^ An example of an account-level material weakness disclosure in the *FairPoint Communications, Inc.* 12/31/2007 10-K reads:

… management determined that our internal control over financial reporting was not effective as of December 31, 2007 because the following material weakness in internal control over financial reporting existed as of December 31, 2007: Our management oversight and review procedures designed to monitor the effectiveness of control activities in the northern New England division were ineffective. As a result, errors existed in capitalized software costs, operating expenses, accounts receivable, prepaid expenses, accounts payable, and accrued expenses in our preliminary 2007 consolidated financial statements.

In contrast, entity-level material weaknesses exist when management’s assessment of internal controls identifies non-accounting application failures. For example, the Audit Analytics category *Segregation of duties/design of controls* indicates problems “associated with the design and use of personnel within an organization. It primarily deals with segregation of duty issues, such as clerks having access to both the cash receipts and the bank reconciliation. It may also deal with more sophisticated design of control issues relating to executives having the ability to change customer records, etc.” An example of an entity-level material weakness disclosure from the *Alphatec Holdings, Inc.* 06/30/2006 10-Q reads:

Our independent registered public accounting firm advised our board of directors and our management that our process for our financial statement year-end close and reporting was insufficiently defined and represented a deficiency in the design and operating effectiveness of our year-end close and reporting controls. One of the primary causes of the deficiency in the financial statement close and reporting process noted by our independent registered public accounting firm was the inadequate staffing in our financial accounting and reporting functions.

In summary, account-level material weaknesses relate to account balances and transaction-level processes, whereas entity-level material weaknesses relate to control and financial reporting processes in general. These latter types of weaknesses pose a serious concern regarding management’s ability to prepare financial reports that fairly reflect the results of operations (Doyle et al., 2007).

We collect material weaknesses in disclosure controls and procedures for our sample companies on a quarterly basis. We use these data to create discrete variables, *MW_ALL_n_m*, that measure the number of material weaknesses, as reported in the Audit Analytics 302 database, disclosed in quarters *n* through *m* following the IPO. We also collect the details necessary to categorize material weaknesses as account-level (i.e., accounting-related) weaknesses or entity-level weaknesses. *MW_ACC_n_m* measures the number of account-level material weaknesses disclosed in quarters *n* through *m*, and *MW_ENTITY_n_m* measures the number of entity-level material weaknesses disclosed in quarters *n* through *m*.

**Fig. 1:**
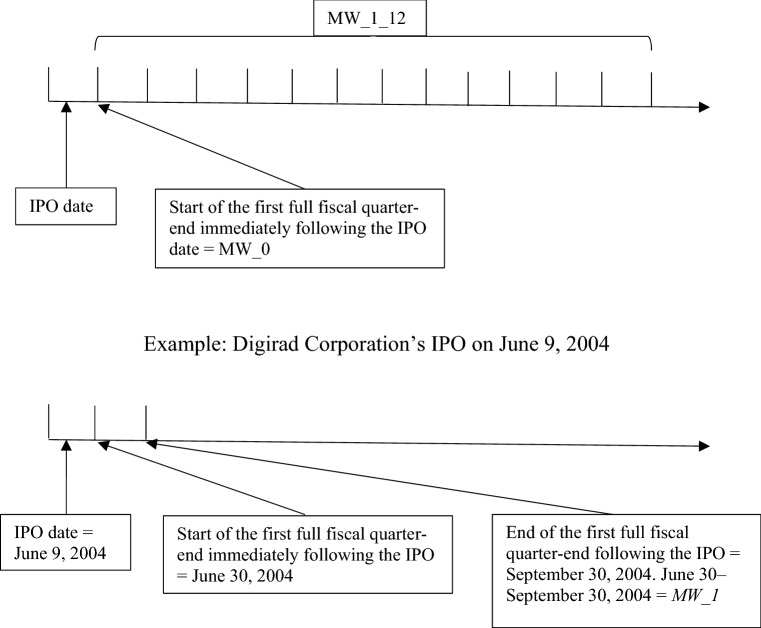
Timeline for the measurement of material weaknesses in disclosure controls and procedures for sample IPO companies. This figure illustrates the timing of material weakness disclosures for our sample of IPO companies. *MW_1* is the first full quarter after the company goes public, *MW_2* is the second full quarter, etc. *MW_ALL_1_8* measures the number of material weaknesses disclosed in the first 8 (full) quarters following the IPO, and *MW_ALL_1_12* measures the number of material weaknesses disclosed in the first 12 (full) quarters following the IPO

In Fig. 1, we present a timeline of the material weakness disclosures post-IPO. We label the end date of the first fiscal quarter immediately following the IPO date “quarter zero” (*MW_0*). *MW_1* is the first full quarter after the company goes public, *MW_2* is the second quarter, and so on. Therefore, *MW_ALL_1_8* measures the number of material weaknesses disclosed over the first 8 (full) quarters following the IPO and *MW_ALL_1_12* measures the number of material weaknesses disclosed over the first 12 (full) quarters following the IPO.^12^

**Fig. 2:**
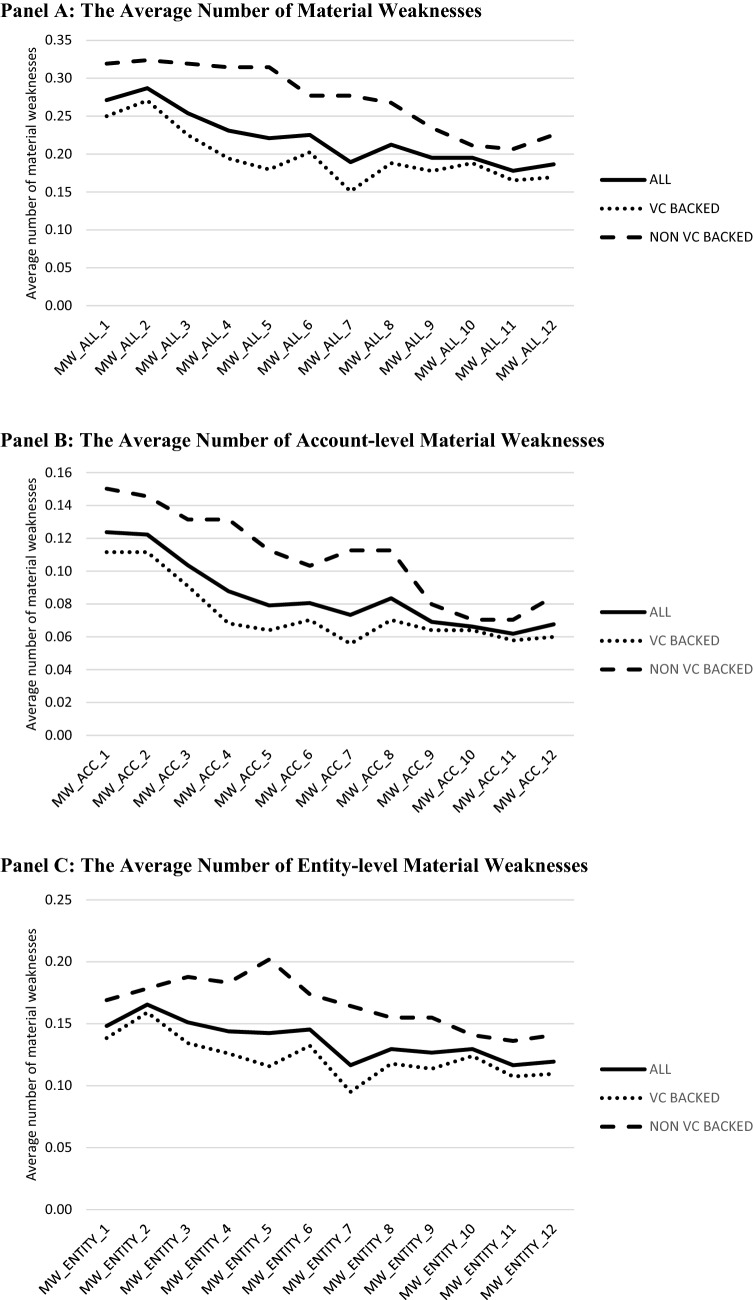
The average number of material weaknesses in disclosure controls and procedures by VC backing. This figure presents the average number of material weaknesses in disclosure controls and procedures (**A**), account-level material weaknesses (**B**), and entity-level material weaknesses (**C**) for all sample companies and for companies backed by VC firms versus not backed by VC firms in fiscal quarters 1 through 12 following the IPO

In Fig. 2, Panel A, we present the average number of material weaknesses disclosed by each of our sample companies in quarters 1 through 12 post-IPO. In all quarters, VC-backed companies disclose fewer material weaknesses than do non-VC-backed companies. Panels B and C present the average number of account-level and entity-level material weaknesses, respectively, in quarters 1 through 12 post-IPO. As was the case for all material weaknesses, in all quarters, VC-backed companies disclose fewer account-level and entity-level material weaknesses.

#### Venture Capital

Our primary test variable is $VC{\text{-}}BACKED$, which is an indicator variable set to one if the sample company was backed by a VC fund at the time of its IPO, and zero otherwise. To form this variable, we follow Wongsunwai (2013) and collect data from SDC Thomson One and VentureXpert. We also hand collect the proportion of the company financed by VC funds pre-IPO from the IPO prospectus; this proportion forms our test variable $VC{\text{-}}SHARE$.

#### Control Variables

Following the IPO literature (e.g., Beatty & Ritter, 1986; Loughran & Ritter, 2004), we control for the underwriter’s reputation, *UNDER-REP*, using the Carter-Manaster reputation score, as modified by Loughran and Ritter (2004).^13^ We control for IPO underpricing, *UNDERPRICING*, because it is an important feature in prior IPO research. We control for the sample company’s growth opportunities using the market-to-book ratio, *M/B*, and we control for whether the company made seasoned equity offerings within two years of the IPO, *SEO*.

Our models also include company characteristics that could be related to material weaknesses in disclosure controls and procedures. Following internal control research (e.g., Cheng et al., 2013; Doyle et al., 2007), we control for company size (*MKTCAP*) and complexity, where complexity is proxied for by the existence of a foreign currency adjustment (*FOREIGN*). We also control for the company’s financial performance, using an indicator variable for losses (*LOSS*) and the Altman (1968) measure of bankruptcy risk (*ZSCORE*), because the implementation and maintenance of good internal control systems requires substantial financial resources. Because the strength of disclosure controls and procedures might be influenced by rapid expansion or company restructuring, we follow Cheng et al. (2013) and control for rapid expansion (*GROWTH*) using an indicator variable set to one if the company’s year-over-year sales growth is in the highest quintile of sales growth in the industry, and zero otherwise. We also control for restructurings (*RESTRUCTURING*). In addition, we allow for the possibility that auditor type influences the likelihood of material weaknesses. We follow Doyle et al. (2007) and include a control variable, *AUDITOR*, set to one if the company engages a Big Four audit firm in the IPO year, and zero otherwise.

Finally, we include fixed effects which control for the average effects of industry, IPO year, company location, and VC location (Gompers & Lerner, 1999). We present formal variable definitions and details about the measurement and data sources in the appendix.

### Research Design

We follow prior research on the effects of VC backing (e.g., Hochberg, 2012; Lee & Masulis, 2011; Wongsunwai, 2013) and employ three different econometric procedures to examine the effect of venture capital on material weaknesses in disclosure controls and procedures. First, we estimate a Poisson regression using our pooled sample. Second, we estimate an instrumental variables model. Finally, we estimate a model using a propensity score matched control sample.

#### Using Poisson Regression

Poisson regression is suitable for our main tests because our dependent variable, *MW_ALL_n_m*, is a count variable that takes only discrete, non-negative values. We regress the number of material weaknesses in disclosure controls and procedures in quarters *n* through *m* following the company’s IPO on VC backing and company characteristics as follows:

1 $$M{W}_{AL{L}_{{n}_{m}}}=\alpha +{\varvec{\theta}}VC-BACKED+{\varvec{\beta}}Controls+IFE+TFE+RFE+ \varepsilon $$

where $VC{\text{-}}BACKED$ is an indicator variable set to one if the company was backed by VC investors, and zero otherwise; *Controls* is a vector of company characteristics; and *IFE*, *TFE*, and *RFE* are industry fixed effects, time (year) fixed effects, and region fixed effects (based on the states where the company and the VC firm are headquartered), respectively. By eliminating variation related to industry, year, and region, we better capture the effect of VC backing on the strength of internal controls.

#### Potential Concerns Due to Non-random Assignment

A potential concern that arises from our research design is possible endogeneity because the set of portfolio companies in which VCs invest is not random. Specifically, VCs might select the portfolio companies with better disclosure in place, making it difficult to disentangle selection and monitoring effects. Also, although unlikely, internal control disclosure could be affected by the same characteristics that affect VC selection. We mitigate the concern that results from the regression tests that do not correct for the endogenous nature of the VC selection process might be biased by using instrumental variables regression and matched sample tests, based on propensity score.

#### Using Instrumental Variables Regression

To employ a two-stage instrumental variables regression, in the first stage, we regress VC backing on an instrumental variable and on all other covariates from Eq. (1). In the second stage, we regress the dependent variable—which is an indicator variable set to one if a company disclosed a material weakness in disclosure controls and procedures in the first two or three years following the IPO (depending on the specification), and zero otherwise—on the fitted values of VC backing as calculated from the first-stage regression (*VC-BACKED-FV*) and on all controls. A suitable instrument will affect VC backing but not the strength of disclosure controls and procedures (Larcker & Rusticus, 2010). Our instrument, which is based on a variable that identifies mimicking behavior (Cumming et al., 2019), is the amount of funds invested by VC firms in the state in which the portfolio firm is headquartered three years prior to the IPO (*VC-STATE-INV*). Because the length of time from a VC’s investment to the IPO averages three years (Gompers & Lerner, 1999), this variable proxies for the amount of funding available for start-ups. All else equal, the higher the supply of VC funding, the more likely a company is to receive VC backing, but it is unlikely that the supply of VC funding in the state affects the disclosure controls of the companies in a specific VC’s portfolio. Moreover, instrumental variables related to the supply of venture capital when entrepreneurial companies are founded are unlikely to be related to governance structures years later at the time of the IPO (Hochberg, 2012). Therefore, there is no reason to assume that the general supply of VC funding would have a direct effect on the strength of the investee company’s internal controls years later.

We confirm (in the first column of Table 5) that *VC-STATE-INV* has explanatory power for predicting VC backing (i.e., the coefficient on our instrumental variable in the first-stage regression is statistically significant). Moreover, the area under the receiver operator curve (AROC) of this first-stage model is 0.85, suggesting that the model has very good explanatory power (Hosmer & Lemeshow, 2002). Finally, as explained previously, there is no reason to expect that the state-level supply of venture capital should affect the likelihood of investee-level internal control weaknesses. Thus, we posit that the supply of VC funding is a suitable instrumental variable.

#### Using Propensity Score Matching

To perform propensity score matched tests, we follow guidance in Rosenbaum and Rubin (1983) and compare the treated companies with a control sample of companies that are similar across all of our covariates other than VC backing. The use of this technique reduces the likelihood that our findings result from functional form misspecification (Shipman et al., 2017). First, we estimate propensity scores for all IPO companies in our sample by estimating a logit model of VC backing on company characteristics and on industry, year, and region indicator variables. We use the calculated propensity scores and nearest neighbor matching to select our control sample and estimate a Poisson regression using the matched sample of VC-backed companies and non-VC-backed companies.

## Empirical Results

### Descriptive Statistics

In Table 2, we present company characteristics for the full sample of VC-backed and non-VC-backed IPO companies, along with the results from t-tests for differences in means. We find that, on average, VC-backed companies report more foreign transactions and more losses, and experience a higher risk of bankruptcy, lower rates of growth, and more restructurings. We also find that the mean number of material weaknesses in disclosure controls and procedures for VC-backed companies is lower than the mean number of material weaknesses for non-VC-backed companies, and univariate differences in means are significant for these unmatched samples for quarters 1–8, but they are not significant for quarters 1–12.

**Table 2:** Summary statistics

	Full sample (*N* = 694)			VC-backed (*N* = 483)			Non-VC-backed (*N* = 211)			Diff. in means	*t *test of difference in means
	Mean	Median	SD	Mean	Median	SD	Mean	Median	SD		
Panel A: Internal control risk variables:											
General											
*MW_ALL_1_8*	1.65	0.00	3.50	1.47	0.00	3.09	2.06	0.00	4.24	0.59	2.11
*MW_ALL_1_12*	2.07	0.00	4.35	1.91	0.00	3.95	2.43	0.00	5.11	0.53	1.51
Account level											
*MW_ACC_1_8*	0.65	0.00	1.67	0.56	0.00	1.47	0.85	0.00	2.04	0.29	2.19
*MW_ACC_1_12*	0.79	0.00	2.05	0.72	0.00	1.88	0.95	0.00	2.38	0.23	1.38
Entity level											
*MW_ENTITY_1_8*	1.01	0.00	1.93	0.91	0.00	1.73	1.21	0.00	2.29	0.30	1.94
*MW_ENTITY_1_12*	1.28	0.00	2.42	1.19	0.00	2.19	1.48	0.00	2.85	0.30	1.54
Panel B: Company-level variables:											
*UNDER-REP*	7.60	8.00	1.86	7.96	9.00	1.51	6.83	8.00	2.26	− 1.12	− 7.87
*UNDERPRICING*	0.13	0.06	0.26	0.16	0.09	0.25	0.06	0.01	0.27	− 0.10	− 4.81
*M/B*	5.22	3.76	26.74	5.72	4.02	30.87	4.13	2.89	14.35	− 1.59	− 0.74
*SEO*	0.40	0.00	0.49	0.41	0.00	0.49	0.38	0.00	0.49	− 0.03	− 0.67
*MKTCAP*	6.05	6.07	1.31	6.13	6.08	1.20	5.88	6.02	1.51	− 0.25	− 2.42
*ROA*	− 0.14	− 0.02	0.44	− 0.16	− 0.06	0.40	− 0.10	0.02	0.50	0.05	1.53
*FOREIGN*	0.26	0.00	0.44	0.28	0.00	0.45	0.21	0.00	0.41	− 0.06	− 1.82
*LOSS*	0.57	1.00	0.50	0.65	1.00	0.48	0.40	0.00	0.49	− 0.25	− 6.35
*ZSCORE*	7.63	4.19	13.31	8.41	5.15	12.58	5.96	3.21	14.65	− 2.44	− 2.30
*GROWTH*	1.57	0.32	11.87	1.51	0.34	9.36	1.69	0.24	16.00	0.18	0.19
*RESTRUCTURING*	0.09	0.00	1.44	0.10	0.00	1.62	0.07	0.00	0.93	− 0.04	− 0.32
*AUDITOR*	0.79	1.00	0.41	0.85	1.00	0.36	0.65	1.00	0.48	− 0.20	− 6.40

This table presents summary statistics for the full sample of 694 IPOs from 2002 through 2018 and for the sample partitioned by VC backing. All variables are as described in the appendix

Table 3 presents the Pearson correlation matrix. Consistent with expectations, material weaknesses in disclosure controls and procedures are negatively correlated with VC backing. Moreover, *UNDERPRICING* and *UNDER-REP* are the only variables that are highly correlated with the other variables (specifically, underwriter reputation (*UNDER-REP*), company size (*MKTCAP*), profitability (*ROA*), and financial distress (*ZSCORE*) are highly correlated with *UNDERPRICING*, and auditor size (*AUDITOR*) is highly correlated with *UNDER-REP*).

**Table 3:** Correlations

	(1)	(2)	(3)	(4)	(5)	(6)	(7)	(8)	(9)	(10)	(11)	(12)	(13)	(14)	(15)
	*MW_ALL_1_8*	*MW_ALL_1_12*	*VC_BACKED*	*UNDER-REP*	*UNDERPRICING*	*M/B*	*SEO*	*MKTCAP*	*ROA*	*FOREIGN*	*LOSS*	*ZSCORE*	*GROWTH*	*RESTRUCTURING*	*AUDITOR*
(1)	1.000	0.815*	− 0.078*	− 0.106*	0.053	0.007	− 0.003	− 0.053	− 0.006	0.045	− 0.017	− 0.021	0.043	− 0.001	− 0.136*
(2)		1.000	− 0.056	− 0.104*	0.033	0.013	0.043	− 0.052	− 0.005	0.049	− 0.035	0.025	0.027	− 0.000	− 0.106*
(3)			1.000	0.282*	0.177*	0.028	0.024	0.089*	− 0.057	0.067	0.231*	0.085*	− 0.007	0.011	0.232*
(4)				1.000	0.256*	− 0.048	− 0.020	0.584*	0.287*	0.131*	− 0.085*	0.038	− 0.070	0.039	0.543*
(5)					1.000	0.008	− 0.059	0.405*	0.177*	0.113*	− 0.007	0.194*	0.012	− 0.019	0.187*
(6)						1.000	0.055	− 0.019	− 0.011	− 0.028	0.026	0.027	0.002	− 0.001	− 0.055
(7)							1.000	0.046	− 0.073*	− 0.036	0.070	0.017	− 0.021	− 0.010	0.008
(8)								1.000	0.341*	0.198*	− 0.247*	0.162*	− 0.063	0.050	0.365*
(9)									1.000	0.090*	− 0.416*	0.273*	− 0.120*	0.018	0.197*
(10)										1.000	− 0.046	− 0.009	− 0.024	0.007	0.043
(11)											1.000	− 0.044	0.082*	− 0.052	− 0.014
(12)												1.000	0.003	− 0.020	0.003
(13)													1.000	− 0.006	− 0.045
(14)														1.000	0.008
(15)															1.000

This table presents Pearson correlations. All variables are as described in the appendix

*Indicates that the correlation is statistically significant at the 5% level or lower

### Main Empirical Results

#### Using Poisson Regression

In Table 4, we estimate the association between material weaknesses in disclosure controls and procedures (*MW_ALL****_****n_m*) and VC backing ($VC{\text{-}}BACKED$). Columns (1) and (2) present the effect of VC backing on the disclosure of material weaknesses during the two (*MW_ALL****_****1_8*) and three years (*MW_ALL_1_12*) following the company’s IPO. In both specifications, we find that VC-backed companies are significantly less likely to report material weaknesses in the (two and three) years post-IPO.^14^

**Table 4:** The relation between VC backing and material weaknesses in internal control

		All MWs		Account-level MWs		Entity-level MWs	
		(1)	(2)	(3)	(4)	(5)	(6)
		Coeff	Coeff	Coeff	Coeff	Coeff	Coeff
$VC{\text{-}}BACKED$		− 0.4045**	− 0.2650**	− 0.4860**	− 0.2877**	− 0.3589**	− 0.2533*
		(− 0.6647)	(− 0.5483)	(− 0.3126)	(− 0.2265)	(− 0.3589)	(− 0.3247)
		[− 2.45]	[− 2.17]	[− 2.39]	[− 2.33]	[− 2.27]	[− 1.87]
*UNDER-REP*		0.0143	− 0.0433	0.0218	− 0.0415	0.0116	− 0.0393
		(0.0236)	(− 0.0895)	(0.0140)	(− 0.0327)	(0.0116)	(− 0.0504)
		[0.27]	[− 0.73]	[0.32]	[− 0.66]	[0.27]	[− 0.67]
*UNDERPRICING*		0.6164**	0.4222**	0.6161*	0.6286***	0.6299***	0.3268**
		(1.0128)	(0.8737)	(0.3963)	(0.4947)	(0.6299)	(0.4190)
		[2.54]	[2.40]	[1.86]	[2.79]	[3.20]	[2.17]
*M/B*		0.0028***	0.0022	0.0031*	0.0025	0.0026***	0.0021
		(0.0045)	(0.0046)	(0.0020)	(0.0019)	(0.0026)	(0.0027)
		[2.93]	[1.49]	[1.68]	[1.19]	[4.02]	[1.55]
*SEO*		− 0.0374	0.2095*	− 0.1245	0.1343	0.0198	0.2574***
		(− 0.0614)	(0.4335)	(− 0.0801)	(0.1057)	(0.0198)	(0.3299)
		[− 0.25]	[1.95]	[− 0.77]	[0.95]	[0.14]	[2.73]
*MKTCAP*		− 0.0383	− 0.0072	− 0.1029	− 0.0899	− 0.0020	0.0337
		(− 0.0629)	(− 0.0149)	(− 0.0662)	(− 0.0707)	(− 0.0020)	(0.0432)
		[− 0.49]	[− 0.10]	[− 1.11]	[− 1.04]	[− 0.03]	[0.48]
*ROA*		0.1036	− 0.0818	0.0712	− 0.1472	0.1236	− 0.0357
		(0.1702)	(− 0.1693)	(0.0458)	(− 0.1159)	(0.1235)	(− 0.0458)
		[0.78]	[− 0.44]	[0.64]	[− 0.81]	[0.80]	[− 0.18]
*FOREIGN*		0.1363	0.1351	0.2659	0.3066	0.0624	0.0353
		(0.2239)	(0.2796)	(0.1710)	(0.2413)	(0.0624)	(0.0452)
		[0.90]	[0.64]	[1.46]	[1.29]	[0.43]	[0.18]
*LOSS*		− 0.0920	− 0.3232*	− 0.1637	− 0.3816	− 0.0517	− 0.2850**
		(− 0.1512)	(− 0.6688)	(− 0.1052)	(− 0.3003)	(− 0.0517)	(− 0.3654)
		[− 0.46]	[− 1.87]	[− 0.60]	[− 1.59]	[− 0.33]	[− 2.01]
*ZSCORE*		− 0.0069	0.0044	− 0.0055	0.0078	− 0.0081	0.0018
		(− 0.0114)	(0.0091)	(− 0.0036)	(0.0062)	(− 0.0081)	(0.0024)
		[− 1.30]	[0.71]	[− 1.15]	[1.33]	[− 1.39]	[0.29]
*GROWTH*		0.0029	0.0077*	0.0018	0.0079	0.0036	0.0074*
		(0.0048)	(0.0159)	(0.0012)	(0.0062)	(0.0036)	(0.0095)
		[0.48]	[1.74]	[0.29]	[1.42]	[0.63]	[1.95]
*RESTRUCTURING*		0.0289	0.0043	0.0137	− 0.0019	0.0406	0.0101
		(0.0475)	(0.0089)	(0.0088)	(− 0.0015)	(0.0406)	(0.0130)
		[0.42]	[0.12]	[0.22]	[− 0.05]	[0.56]	[0.26]
*AUDITOR*		− 0.6022**	− 0.4284*	− 0.7635***	− 0.5926***	− 0.4942**	− 0.3313
		(− 0.9895)	(− 0.8864)	(− 0.4910)	(− 0.4664)	(− 0.4942)	(− 0.4247)
		[− 2.30]	[− 1.92]	[− 2.62]	[− 2.67]	[− 2.03]	[− 1.49]
*N*		694	694	694	694	694	694
Pseudo *R*^2^/*R*^2^		0.1851	0.2090	0.2043	0.2269	0.1542	0.1830
Log likelihood		− 1722.735	− 2010.145	− 835.145	− 983.266	− 1104.322	− 1290.917

This table presents the results from estimating Poisson regressions to test for a relation between VC backing and material weaknesses in internal control. The dependent variable is the number of disclosed material weaknesses in disclosure controls and procedures over the first two years following the IPO in Models (1), (3), and (5) or in the first three years following the IPO in Models (2), (4), and (6). The control variables are defined in the appendix. All models include industry, IPO year, and region fixed effects. Robust standard errors are clustered at the industry level

***, **, and * represent 1%, 5%, and 10% significance levels, respectively, for two-tailed tests. Marginal effects appear in parentheses below the coefficient estimates and *z*-statistics appear in square brackets

To investigate the economic significance of our results, we calculate marginal effects. The values for our main variable of interest (i.e., the number of material weaknesses in internal control) for the two and three years post-IPO are − 0.6647and − 0.5483, respectively. This suggests that companies backed by VC firms report up to two fewer material weaknesses in total than do non-VC-backed companies. The results related to other control variables are consistent with expectations. For example, larger companies and companies audited by a Big Four auditor report fewer material weaknesses following the IPO. In contrast, complex companies, high growth companies, and companies with high market-to-book ratios report more material weaknesses post-IPO.

Next, we investigate the relation between VC backing ($VC{\text{-}}BACKED$) and the number of account-level material weaknesses in disclosure controls and procedures (*MW_ACC_n_m*)—in Columns (3) and (4)—and the number of entity-level material weaknesses in disclosure controls and procedures (*MW_ENTITY_n_m*)—in Columns (5) and (6)—in the two and three years post-IPO. We find that post-IPO, companies backed by VC firms disclose, on average, up to one fewer account-level material weaknesses and up to one fewer entity-level material weaknesses than do non-VC-backed companies.^15^

To investigate whether the construction of our material weaknesses variable affects our inferences, in untabulated analyses, we perform tests using the average number of material weaknesses (rather than the sum) in the two and three years post-IPO and obtain similar inferences. We also analyze whether our inferences hold when we use an indicator variable set to one if the company reports *any* material weaknesses disclosure controls and procedures, and zero otherwise. Again, we find that VC-backed companies are less likely to report material weaknesses in disclosure controls and procedures than are non-VC-backed companies.

#### Using Instrumental Variables Regression

To address the concern that inferences arise simply because VC firms invest in companies that already have stronger internal controls, we use the instrumental variable approach discussed previously. Here, we find that VC backing is strongly related to inflows of VC funding in the state. The F-statistic (of 210.29) exceeds the critical value (of 11.59), suggesting that our instrument is effective (Larcker & Rusticus, 2010). In addition, the over-identification test fails to reject the null hypothesis that the instrument is not correlated with the second-stage regression residuals (J-statistic = 6.60, *p* = 0.2518).^16^ This suggests that our instrument is both effective and valid.

We present the results from estimating the second-stage model in Table 5. Here, we find that the coefficients are statistically significant in the two (three) years post-IPO. These results again suggest that VC backing leads to a focus on stronger internal controls, especially for more general entity-level controls, including the separation of duties and tone at the top.

**Table 5:** The relation between VC backing and material weaknesses in internal control using an instrumental variable approach

	First stage model	All MWs		Account-level MWs		Entity-level MWs	
		(1)	(2)	(3)	(4)	(5)	(6)
	Coeff	Coeff	Coeff	Coeff	Coeff	Coeff	Coeff
*VC-BACKED-FV*		− 0.3417***	− 0.2231*	− 0.2684**	− 0.2053	− 0.3417***	− 0.2231*
		[− 2.68]	[− 1.95]	[− 2.05]	[− 1.52]	[− 2.68]	[− 1.95]
*UNDER-REP*	0.3799***	0.0327*	0.0199	0.0247	0.0182	0.0327*	0.0199
	[7.63]	[1.65]	[0.95]	[1.60]	[1.06]	[1.65]	[0.95]
*UNDERPRICING*	1.5109***	0.1206**	0.0976*	0.1312***	0.1084***	0.1206**	0.0976*
	[4.81]	[2.18]	[1.82]	[4.43]	[3.35]	[2.18]	[1.82]
*M/B*	0.0041**	0.0000	− 0.0001	0.0002	0.0002	0.0000	− 0.0001
	[2.06]	[0.08]	[− 0.28]	[0.48]	[0.61]	[0.08]	[− 0.28]
*SEO*	0.2563	0.0406	0.0852***	− 0.0367	0.0050	0.0406	0.0852***
	[1.34]	[1.21]	[3.05]	[− 1.38]	[0.21]	[1.21]	[3.05]
*MKTCAP*	− 0.2548***	0.0029	0.0139	− 0.0308**	− 0.0305***	0.0029	0.0139
	[− 3.17]	[0.12]	[0.51]	[− 2.55]	[− 2.67]	[0.12]	[0.51]
*ROA*	− 0.3261	0.0398	0.0382	0.0080	0.0049	0.0398	0.0382
	[− 1.05]	[0.89]	[0.87]	[0.25]	[0.13]	[0.89]	[0.87]
*FOREIGN*	0.0151	0.0367	0.0338	0.0537***	0.0658***	0.0367	0.0338
	[0.05]	[1.00]	[1.46]	[4.56]	[2.59]	[1.00]	[1.46]
*LOSS*	0.5152	0.0095	− 0.0419	0.0023	− 0.0156	0.0095	− 0.0419
	[1.61]	[0.25]	[− 1.26]	[0.05]	[− 0.41]	[0.25]	[− 1.26]
*ZSCORE*	0.0127	− 0.0012	− 0.0001	0.0003	0.0021	− 0.0012	− 0.0001
	[1.36]	[− 0.98]	[− 0.06]	[0.22]	[1.01]	[− 0.98]	[− 0.06]
*GROWTH*	− 0.0054	0.0004	0.0006	0.0010	0.0011	0.0004	0.0006
	[− 0.74]	[0.31]	[0.45]	[0.61]	[0.77]	[0.31]	[0.45]
*RESTRUCTURING*	0.0931*	0.0194**	0.0158**	0.0158**	0.0111*	0.0194**	0.0158**
	[1.69]	[2.22]	[2.25]	[2.25]	[1.69]	[2.22]	[2.25]
*AUDITOR*	0.7003**	− 0.0511	− 0.0432	− 0.0765	− 0.0335	− 0.0511	− 0.0432
	[2.25]	[− 1.14]	[− 0.85]	[− 1.61]	[− 0.74]	[− 1.14]	[− 0.85]
*VC-STATE-INV*	0.0354***						
	[2.89]						
*N*	694	694	694	694	694	694	694
Pseudo *R*^2^/*R*^2^	0.2964	0.017	0.098	0.013	0.048	0.017	0.098
AROC	0.85						

This table presents the results from estimating the relation between VC backing and material weaknesses in internal control using an instrumental variable approach. $VC{\text{-}}BACKED$ is the dependent variable in the first-stage logit regression. The instrumental variable is *VC-STATE-INV*. In the second-stage OLS regression, we include the fitted values from the first stage (*VC-BACKED-FV*), and the dependent variable is an indicator variable set to one, if a company disclosed material weaknesses in disclosure controls and procedures in the first two years following the IPO in Models (1), (3), and (5) or in the first three years following the IPO in Models (2), (4), and (6), and zero otherwise. The control variables are defined in the appendix. All models include industry, IPO year, and region fixed effects. Robust standard errors are clustered at the industry level

***, **, and * represent 1%, 5%, and 10% significance levels, respectively, for two-tailed tests, and *z*-statistics appear in square brackets below the coefficient estimates

Finally, to overcome any concerns related to fact that the instrument is based on the funding available at the state level, we propose an alternative approach. Following Cumming et al. (2019), we control for the endogeneity of VC backing by instrumenting this with a mimicking variable, *VC-IND-INV*, measured for each investee company as the average funding available in the same industry three years prior to the IPO. Untabulated results again confirm that VC-backed companies have stronger internal controls.^17^

#### Using Propensity Score Matching

In Table 6, we report the results from estimating Eq. (1) using the propensity score matched samples of VC-backed and non-VC-backed IPO companies.^18^ Again, we find significantly fewer material weaknesses, both overall and at the entity and account levels, in the two and three years post-IPO, when companies are backed by VC firms. These results once again support our expectation that VC backing improves the quality of the internal control environment. However, in all analyses, we do not find improved internal control quality when VC backing is from VC firms with higher reputations. This finding suggests that the quality of internal controls is important to VC firms regardless of their modified Carter-Manaster reputation score.

**Table 6:** The relation between VC backing and material weaknesses in internal control using a matched sample approach

	All MWs		Account-level MWs		Entity-level MWs		
	(1)	(2)	(3)	(4)	(5)		(6)
	Coeff	Coeff	Coeff	Coeff	Coeff		Coeff
$VC-BACKED$	− 0.4936**	− 0.3107*	− 0.6383**	− 0.3956*	− 0.4160**		− 0.2736*
	(− 0.8627)	(− 0.6735)	(− 0.4425)	(− 0.3256)	(− 0.4385		(− 0.3678)
	[− 2.32]	[− 1.84]	[− 2.39]	[− 1.80]	[− 2.22]		[− 1.72]
*UNDER− REP*	0.0671	0.0523	0.0703	0.0317	0.0694		0.0603
	(0.1173)	(0.1135)	(0.0487)	(0.0261)	(0.0732)		(0.0811)
	[0.86]	[0.61]	[0.61]	[0.26]	[1.17]		[0.80]
*UNDERPRICING*	0.9719**	0.5573**	0.7552	0.6617*	1.0907***		0.5511**
	(1.6985)	(1.2080)	(0.5236)	(0.5446)	(1.1498)		(0.7408)
	[2.46]	[2.15]	[1.49]	[1.67]	[2.92]		[2.14]
*M/B*	0.0094**	0.0140***	0.0066	0.0108*	0.0112***		0.0156***
	(0.0165)	(0.0303)	(0.0046)	(0.0089)	(0.0119)		(0.0209)
	[2.54]	[3.80]	[1.09]	[1.79]	[3.95]		[5.15]
*SEO*	0.0316	0.2097	− 0.0491	0.1857	0.0790		0.2440
	(0.0553)	(0.4545)	(− 0.0340)	(0.1528)	(0.0833)		(0.3280)
	[0.15]	[1.17]	[− 0.18]	[0.68]	[0.43]		[1.63]
*MKTCAP*	− 0.0824	− 0.1379	− 0.1149	− 0.1953*	− 0.0643		− 0.1065
	(− 0.1440)	(− 0.2989)	(− 0.0796)	(− 0.1607)	(− 0.0677)		(− 0.1432)
	[− 0.89]	[− 1.40]	[− 0.98]	[− 1.65]	[− 0.75]		[− 1.10]
*ROA*	− 0.2125	− 0.2810***	− 0.2442*	− 0.3479***	− 0.2010		− 0.2496***
	(− 0.3714)	(− 0.6090)	(− 0.1693)	(− 0.2863)	(− 0.2119)		(− 0.3355)
	[− 1.62]	[− 3.13]	[− 1.74]	[− 3.36]	[− 1.49]		[− 2.67]
*FOREIGN*	0.0732	0.0780	0.0802	0.2292	0.0739		0.0063
	(0.1280)	(0.1691)	(0.0556)	(0.1887)	(0.0779)		(0.0084)
	[0.36]	[0.33]	[0.29]	[0.77]	[0.43]		[0.03]
*LOSS*	− 0.0139	− 0.2759	− 0.0483	− 0.3124	− 0.0061		− 0.2535*
	(− 0.0243)	(− 0.5980)	(− 0.0335)	(− 0.2571)	(− 0.0065)		(− 0.3408)
	[− 0.05]	[− 1.45]	[− 0.13]	[− 1.08]	[− 0.03]		[− 1.74]
*ZSCORE*	0.0023	0.0137***	0.0043	0.0224***	0.0010		0.0096***
	(0.0041)	(0.0298)	(0.0030)	(0.0185)	(0.0010)		(0.0130)
	[0.29]	[3.82]	[0.43]	[3.89]	[0.13]		[2.80]
*GROWTH*	0.0149***	0.0126***	0.0195***	0.0170***	0.0123***		0.0104***
	(0.0260)	(0.0272)	(0.0135)	(0.0140)	(0.0130)		(0.0140)
	[4.48]	[4.34]	[4.90]	[5.08]	[3.74]		[3.59]
*RESTRUCTURING*	0.0978	− 0.0541	0.0954	− 0.3430	0.1059		0.0041
	(0.1709)	(− 0.1172)	(0.0662)	(− 0.2823)	(0.1116)		(0.0055)
	[0.72]	[− 0.39]	[0.63]	[− 1.19]	[0.86]		[0.04]
*AUDITOR*	− 0.4568*	− 0.1914	− 0.6339*	− 0.3512	− 0.3436		− 0.0945
	(− 0.7984)	(− 0.4149)	(− 0.4395)	(− 0.2891)	(− 0.3623)		(− 0.1270)
	[− 1.77]	[− 0.98]	[− 1.82]	[− 1.44]	[− 1.57]		[− 0.50]
*N*	424	424	424	424	424		424
Pseudo *R*^2^/*R*^2^	0.2724	0.3077	0.3116	0.3470	0.2245		0.2630
Log likelihood	− 955.041	− 1090.096	− 462.042	− 514.148	− 621.218		− 719.807

This table presents results from estimating Poisson regressions to test for a relation between VC backing and material weaknesses in internal control using a matched sample approach. We match VC-backed and non-VC-backed companies using propensity score matching and the nearest neighbor approach. The matching variables are *UNDER-REP*, *UNDERPRICING*, *M/B*, *SEO*, *MKTCAP*, *ROA*, *FOREIGN*, *LOSS*, *ZSCORE*, *GROWTH*, *RESTRUCTURING*, *AUDITOR,* year, and industry. The dependent variable is the number of disclosed material weaknesses in disclosure controls and procedures over the first two years following the IPO in Models (1), (3), and (5) or in the first three years following the IPO in Models (2), (4), and (6). We present the results for all material weaknesses in Columns (1) and (2), account-level material weaknesses in Columns (3) and (4), and entity-level material weaknesses in Columns (5) and (6). The control variables are defined in the appendix. All models include industry, IPO year, and region fixed effects. Robust standard errors are clustered at the industry level

***, **, and * represent 1%, 5%, and 10% significance levels, respectively, using two-tailed tests. Marginal effects appear in parentheses below the coefficient estimates, and *z*-statistics appear in square brackets

Although the analyses presented above mitigate problems related to endogeneity and selection, we are not able to document the direct channels through which VC firms affect the quality of disclosure. However, based on findings in Kaplan and Strömberg (2003) and Bernstein et al. (2016), among others, we can deduce that because of their close relationships with their portfolio companies, VC firms affect disclosure via on-site involvement as well as by exercising their controlling cash flow rights, board rights, voting rights, liquidation rights, and other control rights.^19^

### Additional Analyses

In additional analyses, we investigate whether the strength of disclosure controls and procedures varies with the percentage of shares held by VC firms, as well as whether material weaknesses are more informative about financial reporting quality when companies have VC backing.

#### Material Weaknesses and VC Shareholdings

To investigate whether the strength of disclosure controls and procedures varies with the percentage of shares held by VC firms, we use hand-collected data on VC shareholdings from IPO prospectuses. Our sample for this test consists of all IPOs from 2002 through 2010 that meet our selection criteria.

In Table 7, we report results from tests for a relation between material weaknesses (all, account level, and entity level) and VC shareholdings. We find a negative and statistically significant association between VC shareholdings and all types of material weaknesses in disclosure controls and procedures. Specifically, a 1% increase in VC shareholdings decreases the average number of material weaknesses by 1.79 (1.43)% in the two (three) years following the IPO. Similarly, a 1% increase in VC shareholdings decreases the average number of account-level material weaknesses by 1.09 (0.65)% and decreases the average number of entity-level material weaknesses by 0.78 (0.75)%. Therefore, we conclude that increased VC backing leads to an improvement in the control environment of newly public companies.

**Table 7:** The relation between VC shareholdings and material weaknesses in internal control

	All MWs		Account-level MWs		Entity-level MWs	
	(1)	(2)	(3)	(4)	(5)	(6)
	Coeff	Coeff	Coeff	Coeff	Coeff	Coeff
*VC-SHARE*	− 0.0112**	− 0.0066**	− 0.0173***	− 0.0077**	− 0.0081*	− 0.0057**
	(− 0.0179)	(− 0.0143)	(− 0.0109)	(− 0.0065)	(− 0.0078)	(− 0.0075)
	[− 2.13]	[− 2.44]	[− 2.63]	[− 1.98]	[− 1.69]	[− 2.36]
*UNDER-REP*	(− 0.0237)	0.0389	− 0.0111	0.0181	− 0.0131	0.0558
	− 0.0378	(0.0839)	(− 0.0070)	(0.0152)	(− 0.0126)	(0.0737)
	[− 0.15]	[0.23]	[− 0.08]	[0.10]	[− 0.08]	[0.35]
*UNDERPRICING*	1.0640*	1.2028**	1.0606	0.8984	1.0931*	1.3937**
	(1.6968)	(2.5968)	(0.6708)	(0.7521)	(1.0517)	(1.8425)
	[1.85]	[2.12]	[1.62]	[1.29]	[1.76]	[2.47]
*M/B*	− 0.0074	0.0103	− 0.0049	0.0142	− 0.0100	0.0081
	(− 0.0118)	(0.0223)	(− 0.0031)	(0.0118)	(− 0.0096)	(0.0108)
	[− 0.29]	[0.62]	[− 0.19]	[0.80]	[− 0.41]	[0.47]
*SEO*	− 0.3093	− 0.2210*	− 0.2524	− 0.2760	− 0.3426	− 0.1928
	(− 0.4932)	(− 0.4772)	(− 0.1597)	(− 0.2311)	(− 0.3297)	(− 0.2549)
	[− 1.52]	[− 1.70]	[− 0.96]	[− 1.46]	[− 1.51]	[− 1.34]
*MKTCAP*	0.1086	− 0.0578	− 0.0498	− 0.1416	0.1966	− 0.0119
	(0.1731)	(− 0.1247)	(− 0.0315)	(− 0.1186)	(0.1892)	(− 0.0157)
	[0.77]	[− 0.36]	[− 0.31]	[− 0.69]	[1.52]	[− 0.09]
*ROA*	− 0.3047	− 0.4606**	− 0.2574	− 0.4993**	− 0.3467	− 0.4410**
	(− 0.4859)	(− 0.9945)	(− 0.1628)	(− 0.4180)	(− 0.3336)	(− 0.5830)
	[− 1.30]	[− 2.48]	[− 1.06]	[− 2.52]	[− 1.43]	[− 2.53]
*FOREIGN*	0.2373	0.3768	0.6285	0.6237	0.0499	0.2298
	(0.3785)	(0.8135)	(0.3975)	(0.5221)	(0.0480)	(0.3038)
	[0.36]	[0.53]	[0.75]	[0.73]	[0.09]	[0.36]
*LOSS*	0.5145**	0.1692	0.6342**	0.1940	0.4685**	0.1649
	(0.8205)	(0.3653)	(0.4011)	(0.1624)	(0.4508)	(0.2180)
	[2.27]	[1.01]	[2.24]	[0.77]	[2.29]	[1.19]
*ZSCORE*	− 0.0099	− 0.0011	− 0.0078	0.0015	− 0.0108	− 0.0031
	(− 0.0157)	(− 0.0023)	(− 0.0049)	(0.0012)	(− 0.0104)	(− 0.0041)
	[− 1.06]	[− 0.10]	[− 0.72]	[0.11]	[− 1.25]	[− 0.32]
*GROWTH*	0.0159***	0.0107***	0.0213***	0.0135***	0.0129***	0.0086**
	(0.0254)	(0.0230)	(0.0134)	(0.0113)	(0.0124)	(0.0114)
	[3.13]	[2.64]	[3.18]	[3.05]	[2.69]	[2.18]
*RESTRUCTURING*	− 0.4248***	− 0.2548	− 0.1606	− 0.0573	− 0.4203***	− 0.2749*
	(− 0.6775)	(− 0.5502)	(− 0.1016)	(− 0.0480)	(− 0.4044)	(− 0.3634)
	[− 3.14]	[− 1.41]	[− 0.36]	[− 0.12]	[− 3.76]	[− 1.92]
*AUDITOR*	− 0.3253	− 0.0983	− 0.2273	− 0.0187	− 0.3969	− 0.1467
	(− 0.5188)	(− 0.2123)	(− 0.1438)	(− 0.0157)	(− 0.3818)	(− 0.1940)
	[− 1.23]	[− 0.48]	[− 0.99]	[− 0.07]	[− 1.36]	[− 0.61]
*N*	264	264	264	264	264	264
Pseudo *R*^2^/*R*^2^	0.3363	0.2815	0.3678	0.3162	0.2891	0.2435
Log likelihood	− 499.391	− 676.019	− 238.631	− 320.272	− 322.896	− 446.229

This table presents the results from estimating the relation between VC shareholdings and material weaknesses in internal control. The dependent variable is the number of disclosed material weaknesses in disclosure controls and procedures over the first two years following the IPO in Models (1), (3), and (5) or in the first three years following the IPO in Models (2), (4), and (6). The control variables are defined in the appendix. All models include industry, IPO year, and region fixed effects. Robust standard errors are clustered at the industry level

***, **, and * represent 1%, 5%, and 10% significance levels, respectively, for two-tailed tests. Marginal effects appear in parentheses below the coefficient estimates, and *z*-statistics appear in square brackets

#### VC Backing and the Informativeness of Material Weakness Disclosures

The primary objective of the material weakness disclosure requirements under SOX is to warn market participants about potential accounting problems that could impact the reliability of the financial statements. Therefore, the role of these disclosures is to warn the financial markets about potential problems or fraud within the company, making quality of these disclosures an important ethical issue. Financial statement restatements are an ex post-indication of low financial reporting quality during the years in which related misstatements occurred. Therefore, we investigate whether material weaknesses in disclosure controls and procedures are more informative about the likelihood of current period financial statement misstatements when companies are VC-backed. That is, we test whether material weaknesses serve as a better “early warning signal” for investors when newly public companies are VC-backed. We conjecture that although VC-backed companies report fewer material weaknesses in disclosure controls and procedures, the quality of their internal control reporting should be higher, such that material weaknesses reported by VC-backed companies should be more indicative of financial statement misstatements (which will be revealed by future financial statement restatements).

To test our conjecture, we use Audit Analytics to identify income-decreasing financial statement restatements (which indicate income-increasing misstatements) that occur through 2018. We restrict this test to income-increasing misstatements because we posit that these types of misstatements should be most concerning to market participants, including VC funds, because negative stock price reactions are more likely for accompany income-decreasing restatements (Palmrose et al., 2004).^20^ We find that 19 (22)% of non-VC-backed companies and 15 (17)% of VC-backed companies make misstatements in the two (three) years post-IPO. Once again, this suggests that VC backing improves financial reporting quality.

Next, we test whether the disclosure of material weaknesses in internal control provides an early warning signal about misstatements (as revealed through future financial statement restatements), and whether this varies with VC backing.^21^ In Table 8, we estimate an ordinary least squares (OLS) regression model where the dependent variable, *REST_1_8-D* (*REST_1_12-D*), is an indicator variable set to one if financial statements related to quarters 1 through 8 (1 through 12) are corrected in subsequent restatements, and zero otherwise. In order to capture the effect of VC backing on the likelihood that misstatements are preceded by material weakness disclosures, we interact our main variable of interest, *VC_BACKED*, with an indicator variable, *MW_ALL_1_8_D* (*MW_ALL_1_12_D*), set to one if the company disclosed a material weakness in quarters 1 through 8 (1 through 12), and zero otherwise.

**Table 8:** The effect of VC backing on the relation between material weaknesses in internal control and future financial statement restatements

	*REST-1–8-D*	*REST-1–12-D*
	(1)	(2)
	Coeff.	Coeff.
$VC{\text{-}}BACKED$	− 0.022	− 0.018
	[− 0.75]	[− 0.63]
*MW-ALL-1–8-D*	0.072	
	[1.21]	
$VC{\text{-}}BACKED$× *MW-ALL-1–8-D*	0.164*	
	[2.08]	
*MW-ALL-1–12-D*		0.080
		[1.48]
$VC{\text{-}}BACKED$× *MW-ALL-1–12-D*		0.151*
		[2.28]
CONTROLS	Yes	Yes
Joint tests (*p* values)		
*MW* + $VC{\text{-}}BACKED$ × *MW*	0.0417**	0.0390**
*N*	391	391
*R*^2^	0.180	0.183

This table presents the results from estimating a linear probability model to test whether the relation between material weaknesses in disclosure controls and procedures and future financial statement restatements varies with VC backing. The dependent variable in Model (1) is an indicator variable set to one if misstatements related to quarters 1 through 8 are identified in subsequent financial statement restatements, and zero otherwise, and the dependent variable in Model (2) is an indicator variable set to one if misstatements related to quarters 1 through 12 are identified in subsequent financial statement restatements. The variables are defined in the appendix. All models include control variables, industry, IPO year, and region fixed effects. Robust standard errors are clustered at the industry level

***, **, and * represent 1%, 5%, and 10% significance levels, respectively, for two-tailed tests. *t*-statistics appear in square brackets below the coefficient estimates

For non-VC-backed companies, we do not find a significant relation between the reporting of material weaknesses and financial statement misstatements. In contrast, the joint test on *MW* + $VC{\text{-}}BACKED$*MW is statistically significant at the 5% level, revealing that for VC-backed companies, the disclosure of material weaknesses is positively associated with a future restatement such that material weakness disclosures provide an early warning about the likelihood that financial statements are misstated. Moreover, the interaction term is positive and statistically significant, suggesting that the disclosure of material weaknesses is more informative about financial reporting quality for VC-backed companies than for non-VC-backed companies.

## Conclusion

In this paper, we use management reports of material weaknesses in disclosure controls and procedures mandated under SOX to test whether VC firms affect the disclosures at their investee companies. Our results are important from the business ethics point of view because high-quality disclosure is important for company transparency in financial markets. The quality of the internal control system and of company disclosures are ethical decisions made by managers and these decisions have consequences for future financial reporting transparency and the occurrence of fraud.

Our empirical tests reveal that VC-backed companies have higher-quality disclosure controls and procedures, at the account level and entity level. This means that VC backing is associated with fewer violations of specific accounting rules and with fewer pervasive problems such as poor “tone at the top” and weak separation of duties, both of which can lead to financial reporting fraud. In addition, material weakness disclosures are more informative about financial reporting quality and are more useful in predicting future financial statement restatements, which indicate that the financial statements previously released by managers were materially misstated, when companies are backed by VC firms. This, at least to some extent, shows that the involvement of VCs can help to prevent an anti-disclosure bias in their portfolio companies. The quality of disclosures has important implications for financial statement users and for capital markets overall because high-quality disclosure is essential for trust and efficient and effective capital allocation in society. Overall, the results from our analyses support the notion that VCs stimulate strong internal control systems and informative disclosure by their investee companies. Future research can explore the channels through which VC firms influence their investee companies. For example, it would be interesting to disentangle the impacts of contracting, voting, incentive compensation, and direct monitoring. Although some prior research attempts to document these sorts of effects, they typically require access to proprietary data. Also, future work could explore other regulatory factors (e.g., see Smith et al., 2022) in influencing the quality of material weakness disclosures by VCs.

Finally, as is the case with almost all archival research, our study is subject to potential limitations, some of which may be addressed in future work. For example, our identification strategy is not perfect so future work may be able to use proprietary data to better assess causality in this context. Furthermore, our analyses and inferences rely on a sample of US IPO companies from 2002 through 2018. Future research on the disclosure of material weaknesses in other institutional contexts and time periods could shed further light on the generalizability of our findings and could provide new insights. Finally, although we contribute to the literature by analyzing some ethical issues related to the material weaknesses in disclosure controls and the disclosure of these weaknesses, future research could examine connections between material weaknesses and ethical violations such as different forms of financial misconduct or market manipulation.

## Appendix: Variable Descriptions

**Table:**

Variable	Variable definition [Source]
*MW_ALL_n_m*	The number of material weaknesses in internal control disclosed in quarters *n* through *m* post-IPO [Audit Analytics]
*MW_ACC_n_m*	The number of account-level material weaknesses in internal control disclosed in quarters *n* through *m* post-IPO [Audit Analytics]
*MW_ENTITY_n_m*	The number of entity-level material weaknesses in internal control disclosed in quarters *n* through *m* post-IPO [Audit Analytics]
$VC{\text{-}}BACKED$	An indicator variable set to one if the company was backed by a VC fund, and zero otherwise [Thomson One SDC, VentureXpert]
*UNDER-REP*	The lead underwriter’s Carter-Manaster reputation score as modified by Ritter (2014b) [http://bear.warrington.ufl.edu/ritter/ipodata.htm]
*UNDERPRICING*	The 1st-day raw stock return for IPO issuers [CRSP]
*M/B*	The market-to-book ratio [COMPUSTAT]
*SEO*	An indicator variable set to one if the company made at least one seasoned equity offerings within two years of the IPO, and zero otherwise [Thomson One SDC]
*MKTCAP*	The log of share price times the number of shares outstanding [COMPUSTAT]
*ROA*	Return on assets [COMPUSTAT]
*FOREIGN*	An indicator variable set to one if the company reported a non-zero foreign currency translation in the IPO year or in the prior year, and zero otherwise [COMPUSTAT]
*LOSS*	An indicator variable set to one if earnings before extraordinary items in the IPO year or in the prior year are less than zero, and zero otherwise [COMPUSTAT]
*ZSCORE*	Altman’s Z-score in year t [COMPUSTAT]
*GROWTH*	The change in sales from year * t* − 1 to year *t*, scaled by sales in year * t* − 1 [COMPUSTAT]
*RESTRUCTURING*	The change in aggregate restructuring charges from year * t* − 1 to year *t*, scaled by the aggregate restructuring charges in year *t* − 1 [COMPUSTAT]
*AUDITOR*	An indicator variable set to one if the company engaged a Big Four audit firm (i.e., Deloitte and Touche, EY, KPMG, or PwC) in the IPO year, and zero otherwise [COMPUSTAT]
*VC-STATE-INV*	The amount invested by VC firms in the state in which the company is headquartered three years prior to the IPO [VentureXpert]

## Footnotes

1 For example, Holder-Webb and Cohen (2007), Jo and Kim (2008), and Baudot et al. (2021) consider the effect of disclosure on firm risk and performance, as well as the importance of analyzing disclosure failures in assessing risk. Blanc et al. (2019) provides a case analysis showing that proper disclosure policy is necessary for corporate legitimacy, and Dupont and Karpoff (2020) explains that financial disclosures are a key part of the “trust triangle,” which is comprised of a society’s legal framework, reputational capital, and corporate culture. Finally, Fasterling (2012) identifies conditions necessary for disclosure to serve an ethical role.

2 See, for example, https://www.wealthmanagement.com/news/top-wire-houses-under-investigation-ipo-scandal and https://www.businessinsider.com/facebook-ipo-disclosure-scandal-2012-5?IR=T.

3 According to the Committee on Sponsoring Organizations of the Treadway Commission, internal controls over financial reporting are “a process designed by, or under the supervision of, the registrant’s principal executive and principal financial officers, or persons performing similar functions, and effected by the registrant’s board of directors, management and other personnel, to provide reasonable assurance regarding the reliability of financial reporting and the preparation of financial statements for external purposes in accordance with generally accepted accounting principles” (see http://www.sec.gov/about/laws/soa2002.pdf).

4 A material weakness is “a deficiency, or a combination of deficiencies, in internal control over financial reporting, such that there is a reasonable possibility that a material misstatement of the company’s annual or interim financial statements will not be prevented or detected on a timely basis” (PCAOB 2007, p. 43).

5 According to “How venture capital works” in *Harvard Business Review* (November–December 1998), VC firms spend approximately 40 percent of their time serving as directors and monitors and acting as consultants to their investments; see https://hbr.org/1998/11/how-venture-capital-works. In addition, roundtable discussions on “Venture capital and efficiency of portfolio companies,” summarized in *IIMB Management Review* (December 2010), describe how VC firms contribute to the efficiency of their investee companies; see https://www.sciencedirect.com/science/article/pii/S0970389610000820.

6 For example, Ashbaugh-Skaife et al. (2008) shows that accruals quality improves after the remediation of internal control deficiencies, and Feng et al. (2009) finds that high-quality internal control systems improve the accuracy of management forecasts.

7 Specifically, Kaplan and Strömberg (2003) studies contracts of 14 VC firms with 119 investee companies. The authors find that VC firms hold separate cash flow rights, board rights, voting rights, liquidation rights, and other control rights that are contingent on observable and non-observable performance measures. Their rights to make corporate decisions are provided by board rights and by voting rights, and because the board is generally responsible for hiring, evaluating, and firing top management, as well as advising and ratifying general corporate strategies and decisions, VC firms have considerable influence.

8 See *Guide to Public Company Transformation: Frequently Asked Questions*, available at guide-to-public-company-transformation-third-edition-protiviti_0.pdf. In addition to this reporting requirement, since 2007, Section 404(b) has required that auditors provide an evaluation of the effectiveness of internal controls in the annual report. However, companies with an aggregate worldwide public float of less than $75 million as of the last business day of their most recently completed second fiscal quarter, companies not subject to the Exchange Act reporting requirements for at least 12 calendar months prior, and companies that have not filed at least one annual report are exempt from this requirement (see Rule 12b-2). Moreover, IPO registrants are exempt from Section 404 reporting requirements until their second 10-K filing after the IPO (see *Roadmap for an IPO: A Guide to Going Public*, available at https://www.pwc.com/us/en/deals/publications/assets/pwc-roadmap-for-an-ipo.pdf). Finally, the benefit of using Section 302 data rather than Section 404 data is also that the quarterly data under Section 302 allow for a more precise measure of the timing of the internal control weakness disclosure post-IPO. However, our inferences hold when we replicate our tests using a smaller sample of management and auditor evaluations of internal control quality made under Section 404.

9 See https://www.pwc.com/us/en/services/deals/library/assets/material-weakness-disclosures-in-an-ipo.pdf.

10 See *Sarbanes-Oxley Act of 2002*, available at http://legcounsel.house.gov/Comps/Sarbanes-oxley%20Act%20Of%202002.pdf.

11 Whether capitalizing expenditures in inventory, leaseholds, buildings, or product/software development, developing and applying the appropriate methodology can be difficult and demanding on the internal control system.

12 Our inferences are robust to including the partial quarter immediately following the IPO date.

13 The IPO underwriter reputation rankings are available at http://bear.warrington.ufl.edu/ritter/ipodata.htm.

14 In untabulated analyses, we find a negative, although not statistically significant, difference in the first year post-IPO.

15 The marginal effect for account-level material weaknesses is − 0.3126 (− 0.2265) for the two (three) years post-IPO, and the marginal effect for entity-level material weaknesses is − 0.3589 (− 0.3247) for the two (three) years post- IPO.

16 See Larcker and Rusticus (2010) for a discussion of these tests.

17 These results are available upon request.

18 As explained previously, to calculate the propensity scores, we estimate a logit model for VC backing. Here, we use the following matching variables: *UNDER-REP*, *UNDERPRICING*, *M/B*, *SEO*, *MKTCAP*, *ROA*, *FOREIGN*, *LOSS*, *ZSCORE*, *GROWTH*, *RESTRUCTURING*, *AUDITOR*, as well as year and industry fixed effects.

19 Kaplan and Strömberg (2003) studies contracts of 14 VC firms with 119 investee companies. The authors find that VC firms hold separate cash flow rights, board rights, voting rights, liquidation rights, and other control rights that are contingent on observable and non-observable performance measures. Their rights to make corporate decisions are provided by board rights and by voting rights, and because the board is generally responsible for hiring, evaluating, and firing top management, as well as advising and ratifying general corporate strategies and decisions, VC firms have considerable influence. Kaplan and Strömberg (2003) shows that if the investee performs poorly, VC firms can be granted full control over the company, including firing the management, who are founders in some cases. Therefore, if poor financial disclosures have a significantly negative effect on stock price, VC firms can take control of the company and replace management. Moreover, Bernstein, Giroud, and Townsend (2016) provide evidence that VC firms’ on-site involvement in their portfolio companies increases the likelihood of a successful exit.

20 In untabulated analyses, we find that our inferences are robust to including all misstatements, regardless of their directional effect on income.

21 Because we are interested in whether material weaknesses provide an early warning about financial reporting quality, we eliminate misstatements that occur prior to material weakness disclosures, but our inferences hold regardless.
